# Biochar application ameliorated the nutrient content and fungal community structure in different yellow soil depths in the karst area of Southwest China

**DOI:** 10.3389/fpls.2022.1020832

**Published:** 2022-10-24

**Authors:** Meng Zhang, Yanling Liu, Quanquan Wei, Xiaofeng Gu, Lingling Liu, Jiulan Gou

**Affiliations:** Institute of Soil and Fertilizer, Guizhou Academy of Agricultural Sciences, Guiyang, China

**Keywords:** distiller’s grains biochar, karst yellow soil, soil depth, soil nutrient, fungal community structure

## Abstract

The influence of biochar on the change of nutrient content and fungal community structure is still not clear, especially in different yellow soil depths in karst areas. A soil column leaching simulation experiment was conducted to investigate the influence of biochar on soil content, enzymatic activity, and fungal community diversity and structural composition. Three biochar amounts were studied, namely, 0%(NB, no biochar), 1.0%(LB, low-application-rate biochar), and 4.0% (HB, high-application-rate biochar). The results showed that biochar increased the pH value and the contents of soil organic matter (SOM), total nitrogen (TN), available phosphorus (AP), and available potassium (AK) but reduced the microbial biomass carbon (MBC) and microbial biomass nitrogen (MBN). Furthermore, this effect was enhanced with increasing biochar amount. Biochar was conducive to improving the nutrient availability in topsoil (0–20 cm), especially TN, AK, and MBN. Meanwhile, biochar affected the enzymatic activity, especially the sucrase activity. Biochar affected the diversity and structure of the fungal community, of which HB treatment had the most obvious effect. Among these treatments, *Aspergillus*, *unclassified_Chaetomiaceae*, *Mortierella*, *Spizellomyces*, *Penicillium*, *Fusarium*, and *unclassified_Chromista* fungal genera were the highest. Moreover, biochar inhibited the growth of harmful pathogens and increased the abundance of beneficial fungi in soil, and the effect was enhanced with increasing biochar amount and soil depth. Redundancy analysis (RDA) showed that AK was an important factor in yellow soil, although the main environmental factors affecting the fungal community structure were different in different soil depths. Overall, biochar had a positive effect on improving the land productivity and micro-ecological environment of yellow soil in the karst area.

## 1 Introduction

The karst landform is a kind of geological landscape with special hydrological conditions and geological characteristics. The distribution area of karst landform accounts for about 12.5% of the global land area (Yan et al., 2021; Zhang et al., 2022). The largest concentrated contiguous karst region in the world is located in Southwest China and mainly distributed in China’s provinces such as Guizhou, Yunnan, and Guangxi. A karst ecosystem is very easy to be destroyed, and nutrient leaching is widespread due to shallow soil thickness (Cao et al., 2021; Xiao et al., 2021). These factors are very unfavorable to agricultural production and economic development.

In recent years, biochar is paid great attention as a soil conditioner (Gul et al., 2015; Ali et al., 2022a). The surface of biochar contains the functional groups of –COOH and –OH so that the aqueous solution of biochar is alkaline. Biochar can also increase the pH value of soil and improve the nutrient effectiveness of nitrogen and phosphorus (El-Naggar et al., 2019; Nan et al., 2020). At the same time, biochar has a high ion adsorption exchange performance, which can improve the cation anion exchange capacity and water and fertilizer retention capacity (Baiamonte et al., 2019). Thus, biochar can directly or indirectly participate in the soil nutrient cycling of a farmland ecosystem. Moreover, biochar can affect the transformation and migration of soil nutrients through physical and chemical interactions with soil (Li et al., 2019a). In addition, the abundant pore structure in biochar can provide a habitat for soil microorganisms, which is conducive to their growth and to the improvement of the soil ecological environment (Ji et al., 2019; Song et al., 2022). Thus, biochar has a wide application prospect in soil improvement and ecological restoration areas.

Soil microbes are the main driving factor for soil nutrient cycling and transformation. Soil nutrients and structure can directly or indirectly change the soil microbial abundance and community structure (Ali et al., 2022b). Fungi are an important part of soil microorganisms which play an important role in material circulation, energy transfer, and inhibition of soil-borne diseases (Liu et al., 2021). Fungi can promote the absorption of soil nutrients by the roots and affect the distribution of the soil microbial community (Joergensen and Wichern, 2008). Compared to bacteria, fungi have a more complex life history. When the soil environment changes, fungi can rapidly change their nutrient pattern to defend against an unfavorable external environment; thus, fungi are highly adaptable and have advanced survival strategies (Cheng et al., 2020). Biochar can release nutrients to improve the soil microbial diversity and activity and further affects the soil’s ecological functions (Edenborn et al., 2017). Ibrahim et al. (2020) showed that the combined application of biochar and chemical fertilizer provided more nutrients, which was conducive to stimulating the soil microbial activity and increasing the number of beneficial bacteria. In addition, the carbon skeleton of biochar has good water retention and aeration capacity, which means that biochar can provide a better living environment for the colonization of soil microorganisms (Haider et al., 2022; Ji et al., 2022). Tang et al. (2021) found that biochar could also indirectly affect the soil microbial activity, abundance, and diversity through nutrient uptake or improvement of soil properties. Furthermore, some studies similarly showed that the abundance of soil fungal communities increased with the increase of biochar application, and the effect of biochar on soil microbial diversity was closely related to the timing of biochar application (Yao et al., 2017; Yan et al., 2022). Dong et al. (2017) and Mitchell et al. (2015) showed that the soil microbial community structure did not change when a small amount of biochar was applied to the soil, but it changed significantly when the amount of biochar reached a certain level, indicating that the amount of biochar applied also had a significant effect on the soil microbial community structure. It should be noted that the influence of biochar on fungal diversity is related to the soil types and utilization patterns (Tarin et al., 2021; Zhu et al., 2021). However, few studies have focused on the influence of biochar on soil fungal diversity in karst areas, and the mechanism of biochar on fungal communities in different soil depths is still unclear.

Distiller’s grains are biomass waste generated from the production process of distilled spirits. Distiller’s grains are rich in crude protein and contain varieties of trace elements, vitamins, and yeast, which are not provided by crop straw (Kong et al., 2021). The annual production of distiller’s grains in China exceeds 20 million tons, accounting for 10% in Guizhou Province. However, the actual utilization rate of distiller’s grains is less than 50% per year, and the rest is discarded. Therefore, the comprehensive utilization of wine lees has become a major issue facing the brewing industry. Previous studies found that the application of distiller’s grain biochar significantly altered the bacterial community diversity and community structure of yellow soil in karst areas under natural rainfall conditions (Zhang et al., 2021). Moreover, Kong et al. (2021) indicated that distiller’s grain biochar formed more aromatic ring structures with low H/C and O/C ratios, and showed the best performance for ammonium removal with an adsorption capacity. The present research is based on the short-term study of the application of distiller’s grain biochar in yellow soil of the karst area, with the following research objectives: (i) to study the effects of biochar on soil nutrient content and migration characteristics in different soil depths, (ii) to examine the effects of biochar on the diversity and composition of soil fungal communities, and (iii) to study the effect of biochar on the relationship between soil fungal colonies and soil environmental factors in different soil depths. This research can provide a theoretical framework for the improvement of yellow soil quality and the rational utilization of distiller’s grain resources in karst areas.

## 2 Materials and methods

### 2.1 Site description

The experiment was conducted in the laboratory from July to September in 2019. The tested soil was a typical zonal yellow soil in Guizhou Province, China. The soil samples were collected from the Pingba District (26°22′10.5″N, 106°12′59.1″E) in Anshun City. The basic physical and chemical properties of the soil were as follows: pH of 6.37, soil organic matter (SOM) of 26.80 g·kg^-1^, total nitrogen (TN) of 0.74 g·kg^-1^, available phosphorus (AP) of 48.60 mg·kg^-1^, and available potassium (AK) of 175.0 mg·kg^-1^. Biochar was prepared by oxygen-limited cracking method in the biomass carbonization furnace (SSDP-5000-A, Jiangsu Huaian Huadian Environmental Protection Machinery Manufacturing Co., Ltd., Huaian, China). Appropriately weighted amounts of distiller’s grain were put in the furnace and blown with N_2_ for 5–10 min to exhaust excess air in the furnace. The sample was pyrolyzed at 550°C for 2 h. After cooling, it passed through a 100-mesh sieve and then placed in a brown bottle for the subsequent experiment. The physicochemical properties of the test biochar were as follows: pH of 8.78, organic carbon(OC) of 281.33 g·kg^-1^, TN of 7.49 g·kg^-1^, AP of 1.38 mg·kg^-1^, and AK of 4.62 g·kg^-1^. The characteristic structure of the test biochar was follows: specific surface area (SSA) of 2.12 m^2^·g^-1^, single-point adsorption total pore volume (SPATPV) of 2.95 × 10^-3^ m^3^·g^-1^, and average pore size of 5.55 nm.

### 2.2 Experimental design

The test device was a polyvinyl chloride (PVC) tube with a diameter of 10 cm and height of 50 cm. The bottom of the PVC tube was wrapped with gauze to prevent soil or quartz sand loss. First, the bottom layer of the soil column was filled with 5-cm-thick quartz sand. Then, 4 kg of yellow soil (2 mm) was mixed with 0% (NB, no biochar), 1.0% (LB, low-application-rate biochar), and 4.0% (HB, high-application-rate biochar) of biochar and filled into the PVC tube. The soil on the surface of the soil column was properly pressed to avoid soil spillage. After the soil column was filled, a layer of fine sand was spread on the surface of the column to prevent disturbance of the topsoil by watering. The amount of water added for the first time was 200 ml to saturate the soil column, and then 100 ml of water was added every 4 days. Lastly, the amount and rate of water added in each soil column were kept the same to ensure that the relative soil moisture content was controlled at about 60% of the saturated moisture content. After 60 days of continuous incubation, soil samples were collected and assayed for analysis. Each treatment was repeated three times, and a total of nine samples were counted in the experiment.

### 2.3 Collection of soil samples

At the end of the test, all the PVC tubes were cut into four layers (0–10, 10–20, 20–30, and 30–40 cm). Then, the soil in each layer was mixed well, and soil samples were finally collected. The soil samples were divided into three parts. One portion of each soil sample was wrapped in aluminum foil and quickly packed into centrifuge tubes, frozen in liquid nitrogen, stored at -80°C, and then used later for high-throughput sequencing of associated soil microorganisms. Another portion of each sample was stored at 4°C prior and used to determine the soil microbial biomass carbon (MBC), microbial biomass nitrogen (MBN), and soil enzyme activity. The remaining portion of each sample was air-dried, ground, and sieved for the determination of soil pH, SOM, TN, AP, and AK.

### 2.4 Determination items and methods of analysis

#### 2.4.1 Physical and chemical properties of soil

The physical and chemical properties of soil were determined according to the methods described by Bao (2000). The soil pH value was measured using a 1:2.5 extraction mixture (soil/water, w/v) with a pH meter (FE20K, Mettler Toledo, Zurich, Switzerland). SOM was determined by high-temperature external heating potassium dichromate oxidation volumetric method. TN was metered by H_2_SO_4_–H_2_O_2_ digestion Kjeldahl method. MBC and MBN were acquired by chloroform fumigation–K_2_SO_4_ extraction method. AP was measured by extraction molybdenum antimony anti-colorimetry of 0.5 mol·L^-1^ NaHCO_3_. AK was determined by extraction using ammonium acetate and assessed using a flame photometer (FP640, Shanghai Aopu Analytical Instrument Co., Ltd., Shanghai, China).

#### 2.4.2 Enzyme activities of soil

The urease activities of soil were determined according to the methods described in Bao (2000). Urease activity was determined by incubating 2 g of soil for 24 h at 37°C with urea solution, tris (hydroxymethyl) aminomethane (THAM) buffer, and toluene. Distilled water was added, mixed, and centrifuged at 12,000 pm at 25°C for 10 min, and the supernatant was taken. It was placed at 37°C for 20 min, and the absorbance value was recorded at 578 nm. Sucrase activity was determined according to the method proposed by Yang et al. (2020). The mixture system for assessing soil sucrase activity was obtained by mixing soil with the solution of 8% sucrose at a ratio of 1:3 (w/v), followed by adding 5 ml of phosphate buffer and 5 ml of toluene. After incubation for 24 h in a constant-temperature incubator at 37°C, the supernatant was separated by filtration. The absorbance of the filtrate was determined immediately after at 485 nm using a spectrophotometer (UV-3600i Plus, Shimadzu, Tokyo, Japan). Catalase activity was measured using H_2_O_2_ as the substrate; the mixture was shaken for 20 min, and the filtrate was titrated with 0.1 mol·L^-1^ KMnO_4_. The catalase activity of soil was measured at a wavelength of 480 nm using a spectrophotometer (UV-3600i Plus, Shimadzu, Tokyo, Japan). Denitrification enzyme activity (DEA) assays were carried out based on Bowen et al. (2020) to determine total denitrification (N_2_ + N_2_O) and N_2_O production. Ten grams (dry weight) of soil was weighed into 60-ml serum bottles, and 10 ml of DEA medium (1 mM of KNO_3_, 1 mM of dextrose, and 0.10 mg·L^-1^ of chloramphenicol) was added to each bottle. The bottles were sealed with rubber stoppers and flushed with N_2_ gas for 4 min to remove oxygen. Then, they were equilibrated to atmospheric pressure by inserting through the septum a needle attached to a syringe barrel (with the plunger removed) and filled with water to release excess gas. The bottles were shaken on a reciprocating shaker. Three-milliliter gas samples were taken at 45 and 105 min and stored in vials that had been flushed with N_2_ gas. Nitrous oxide concentration was analyzed on a high-performance gas chromatograph (HP6890, Agilent Palo Alto, California, USA) and configured to allow the back-flushing of samples containing acetylene. The oven temperature was 50°C, and the carrier gas (N_2_) flow rate was 50.8 ml·min^-1^.

#### 2.4.3 Soil DNA extraction and high-throughput sequencing

Fresh soil, equivalent to 0.5 g dry soil weight, was weighed for each sample. FastDNA^®^SPIN Kit for Soil (MP Biomedicals, Shanghai, China) was adopted to extract total DNA from soil microbes; electrophoresis test with 1% agarose was used to determine whether there is degradation or impurity; and a spectrophotometer (Nanodrop 2000, NanoDrop Technology Co., Ltd., Wilmington, Delaware, USA) was used for sample purity. After DNA concentration and purity detection (DNA concentration ≥20 ng·µl^-1^, total amount ≥500 ng; *D*_260/280_ of 1.8–2.0), PCR (ABI GeneAmp R9700, Applied Biosystems, Shanghai, China) was used to amplify the fungal total DNA in the ITS2 region, and the amplification primer used ITS3F (5′-GCATCGATGAAGAACGCAGC-3′) and ITS4R (5′-TCCTCCGCTTATTGATATGC-3′). The amplification procedure was as follows: pre-denaturation at 95°C for 3 min, 27 cycles (denatured at 95°C for 30 s, annealed at 55°C for 30 s, and extended at 72°C for 30 s), then stabilization at 72°C for 10 min, and finally restoration at 4°C. The reaction system of PCR was as follows: 4 μl buffer (5× TransStart FastPfu), 2 μl dNTPs (2.5 mmol·L^-1^), 0.8 μl upstream primer (5 μmol·L^-1^), 0.8 μl downstream primer (5 μmol·L^-1^), 0.4 μl DNA polymerase (TransStart FastPfu), and 10 ng template DNA. Sequencing was performed using the Miseq PE300 platform of Illumina (Shanghai Meiji Biomedical Technology Co., Ltd., Shanghai, China).

#### 2.4.4 Alpha and beta diversity analyses

An operational taxonomic unit (OTU)-based analysis method was used to evaluate the fungal diversities in each sample from each plant (alpha diversity). To estimate the diversity index and species richness (alpha diversity) among the genotypes for each sample, ACE, Chao1, Simpson, and Shannon indices were calculated using the QIIME software (v1.8.0), concerning a sequencing depth of 3%. Statistical analysis was performed using ANOVA with *p*-values to determine the significant differences in the diversity indices or species richness among the plant rhizosphere soil samples. The rarefaction curve and rank abundance curves were calculated at 97% level of similarity of the OTUs.

Beta diversity analysis was adopted for all samples to determine the similarity index of the community structure. At the OTU level of genotypes, beta diversity was calculated using weighted UniFrac distances and was visualized through principal component analysis (PCA).

### 2.5 Statistics and analysis

For statistical calculation, redundancy analysis (RDA) was achieved using the Canoco 4.5 software, and significant relationships between the fungal communities and soil environmental variables were figured out. Single- and multiple-factor analysis of variance (ANOVA) were performed using the SPSS 20.0 software (SPSS Inc., Chicago, IL, USA). The means were separated using the least significant difference (LSD) test at 5% probability level. All figures were drawn using the Origin 8.0 software program (Origin Lab, MA, USA).

## 3 Results

### 3.1 Soil chemical properties

**Figure 1:**
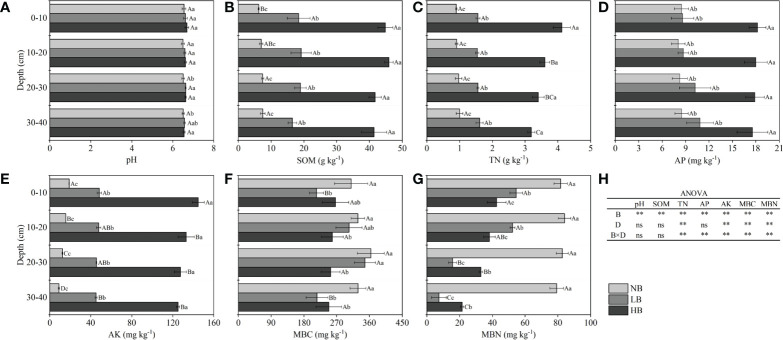
Effects of biochar on **(A)** soil pH, **(B)** soil organic matter (SOM), **(C)** total nitrogen (TN), **(D)** available phosphorus (AP), **(E)** available potassium (AK), **(F)** microbial biomass carbon (MBC), **(G)** microbial biomass nitrogen (MBN), and **(H)** ANOVA. In **(A–G)**, the different capital letters indicate significant differences at *p <*0.05 in different soil depths of the same treatment, and the different lowercase letters indicate significant differences at *p <*0.05 in the same soil depths of different treatments. In **(H)**, B represents treatments of biochar application rate, D represents soil depth, and B × D represents the interaction between biochar application rate and soil depth. ns, no difference; **, statistical significance at *p <*0.01.

The results showed that there were significant differences in the influences of biochar dosage on the pH value and SOM, TN, AP, AK, MBC, and MBN contents at different soil depths (**Figure 1**). Compared with NB treatment, biochar treatments (LB and HB) increased the pH value and SOM, TN, AP, and AK contents by 1.07%–2.15%, 121.64%–621.06%, 60.82%–357.78%, 1.65%–158.80%, and 140.30%–1287.92%, respectively. The MBC and MBN contents decreased by 4.48%–34.25% and 33.24%–90.72%, respectively. Compared with LB treatment, HB treatment increased the pH value and SOM, TN, AP, and AK contents of 0–40 cm by 0.46%–1.06%, 120.49%–151.18%, 98.14%–164.10%, 43.41%–154.59%, and 161.96%–217.91%, respectively. Moreover, the MBN content of 0–20 cm was significantly reduced by 21.89%–26.94% in HB treatment, while it was significantly increased by 110.12%–192.79% in 20–40 cm. Moreover, due to the downward migration of soil nutrients, the AK content for all treatments showed a significant decrease with increasing soil depth. The MBN content for LB treatment and the TN and MBN contents of HB treatment also decreased significantly. The results of the ANOVA showed that the amount of biochar had significant effects on pH, SOM, TN, AP, AK, MBC, and MBN, while soil depth only had significant effects on TN, AK, MBC, and MBN. Moreover, the interaction of the two had significant effects on TN, AP, AK, MBC, and MBN, while there was no effect on pH and SOM.

### 3.2 Soil enzyme activities

**Figure 2:**
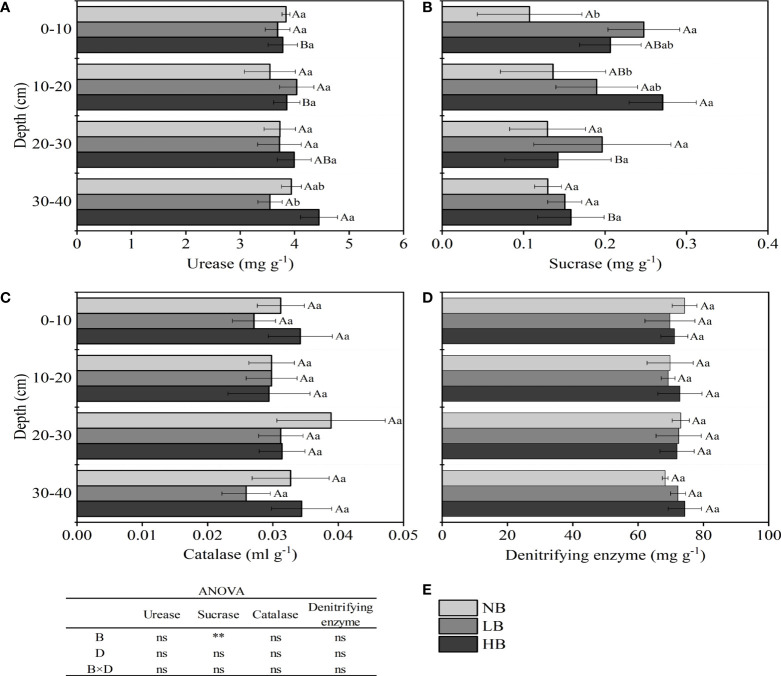
Effects of biochar on **(A)** soil urease, **(B)** sucrase, **(C)** catalase, **(D)** denitrifying enzyme, and **(E)** ANOVA. In **(A–D)**, the different capital letters indicate significant differences at *p <*0.05 in different soil depths of the same treatment, and the different lowercase letters indicate significant differences at *p <*0.05 in the same soil depths of different treatments. In **(E)**, B represents treatments of biochar application rate, D represents soil depth, and B × D represents the interaction between biochar application rate and soil depth; ns, no difference; **, statistical significance at *p <*0.01.

Biochar had little effect on enzyme activity in different soil depths of yellow soil (**Figure 2**). The results of the ANOVA showed that the biochar amount only had a significant effect on sucrose activity, while soil depth and their interaction had no effect on soil enzyme activity. In addition, the urease activity in HB treatment increased with the increase of soil depth, and the urease activity increased by 1.93%–11.41% at 10–40 cm compared with 0–10 cm. Compared with NB treatment, biochar treatments (LB and HB) increased the sucrase activity by 9.96%–130.59% in 0–40 cm, with LB treatment increasing by 130.59% at 0–10 cm and HB treatment increasing by 98.88% at 10–20 cm.

### 3.3 Alpha diversity indexes of the fungal community

Alpha diversity indices were generated for each sample to examine the treatment diversity (**Table 1**). The alpha diversity ACE, Chao 1, Shannon, and Simpson indexes were affected by different treatments, especially in ACE and Simpson indexes. Biochar treatments (LB and HB) had a lower ACE index than NB treatment. The lowest ACE index was recorded in HB treatment (the average value was 361 in 0–40 cm). Similarly, a lower Chao 1 index was observed in LB treatment (the average value was 397 in 0–40 cm) and HB treatment (the average value was 365 in 0–40 cm). Furthermore, the Simpson index was higher in LB treatment (the average value was 0.1468 in 0–40 cm) and HB treatment (the average value was 0.1978 in 0–40 cm) treatment and was lowest (the average value was 0.1309 in 0–40 cm) in NB treatment. However, there was no obvious rule for alpha diversity indexes in different soil depths.

**Table 1:** Effects of biochar on the alpha diversity indexes of the fungal community.

Treatments	Depth (cm)	OTUs	ACE	Chao1	Shannon	Simpson
NB	0–10	285 ± 35 b	339 ± 50 b	334 ± 49 b	2.30 ± 0.21 c	0.2078 ± 0.0488 a
	10–20	389 ± 59 a	478 ± 67 a	471 ± 78 a	3.25 ± 0.18 ab	0.0749 ± 0.0085 b
	20–30	324 ± 55 ab	383 ± 41 ab	381 ± 45 ab	2.62 ± 0.60 bc	0.1668 ± 0.1146 a
	30–40	397 ± 45 a	435 ± 52 ab	438 ± 49 ab	3.34 ± 0.14 a	0.0742 ± 0.0182 b
LB	0–10	361 ± 49 a	383 ± 46 a	390 ± 44 a	3.05 ± 0.68 a	0.1430 ± 0.0414 ab
	10–20	397 ± 50 a	415 ± 51 a	418 ± 54 a	3.16 ± 0.96 a	0.1289 ± 0.0790 b
	20–30	339 ± 15 a	361 ± 14 a	367 ± 8 a	2.50 ± 0.34 a	0.1016 ± 0.0400 b
	30–40	384 ± 51 a	404 ± 61 a	414 ± 60 a	2.69 ± 0.47 a	0.2136 ± 0.1139 a
HB	0–10	289 ± 56 b	328 ± 67 b	339 ± 47 b	2.66 ± 0.23 a	0.1372 ± 0.0725 b
	10–20	300 ± 31 b	337 ± 10 ab	333 ± 20 b	2.93 ± 0.51 a	0.1741 ± 0.1825 b
	20–30	349 ± 17 ab	376 ± 20 ab	377 ± 15 ab	3.05 ± 0.41 a	0.2469 ± 0.0843 a
	30–40	370 ± 20 a	403 ± 21 a	409 ± 11 a	2.71 ± 0.60 a	0.2331 ± 0.1229 a
ANOVA						
B		ns	**	ns	ns	**
D		*	**	*	ns	*
B × D		ns	**	ns	ns	**

The different lowercase letters indicate significant differences at p < 0.05 in different soil depths of the same treatment.

B, treatments of biochar application rate; D, soil depth; B × D, interaction between biochar application rate and soil depth; ns, no difference; *, statistical significance at p <0.05; **, and statistical significance at p <0.01.

### 3.4 Beta diversity of the fungal community structure

**Figure 3:**
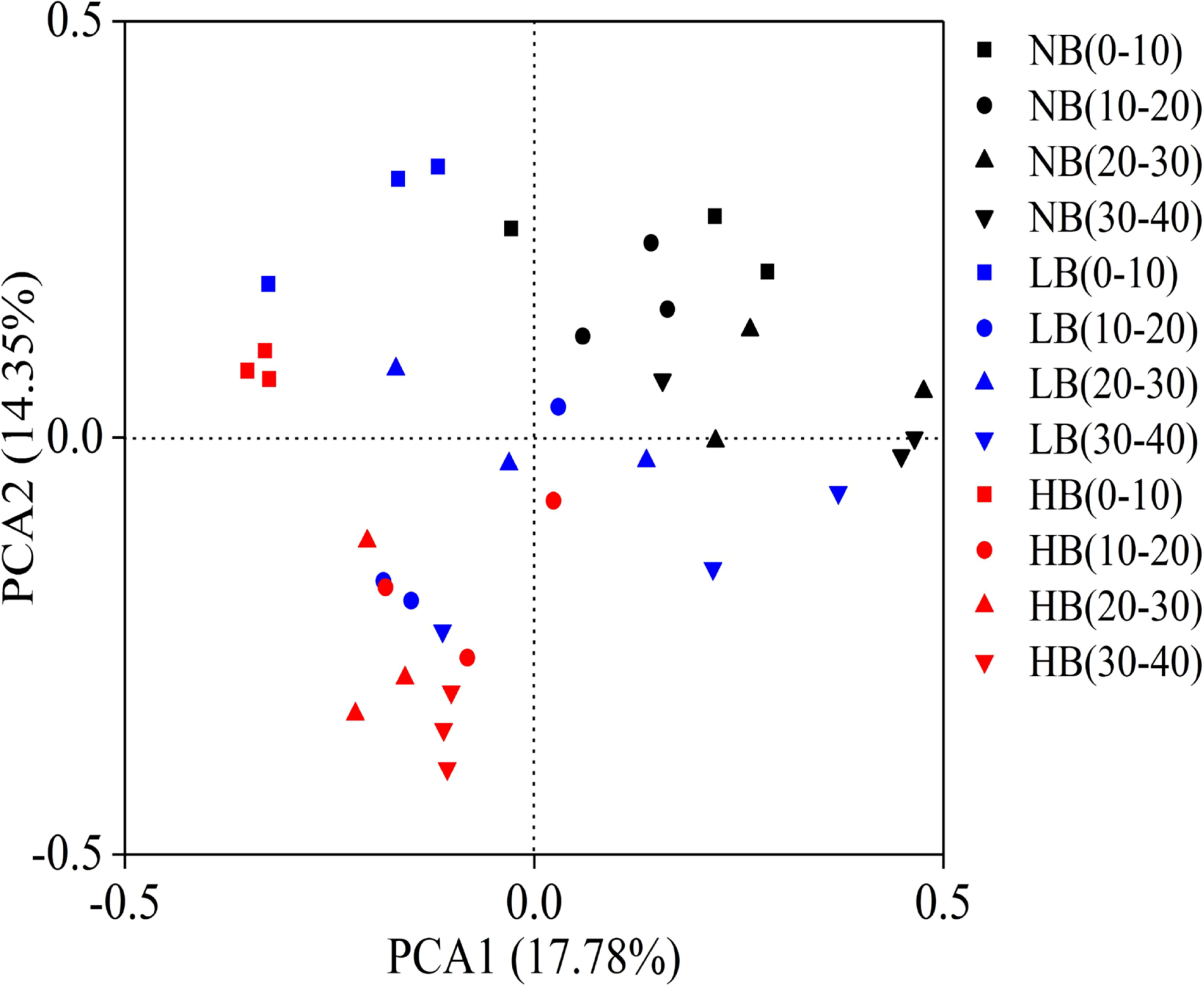
Principal component analysis of the fungal community structure.

PCA was used to assess the similarity and dissimilarity for fungal beta diversity among the treatments. According to the results, the point of NB treatment had a close distance with that of LB treatment, but it was far from that of HB treatment (**Figure 3**). The results indicated that biochar had a significant effect on the fungal community structure, and the degree of effect was closely related to the biochar amount. At the same time, the points were relatively close to each other and even overlapped between LB and HB treatments, which indicated a high coefficient of species similarity in both treatments. It can be understood that the application of biochar can promote the growth of the same species in the composition of soil fungal community.

### 3.5 Community structure and the relative abundance of fungi

**Figure 4:**
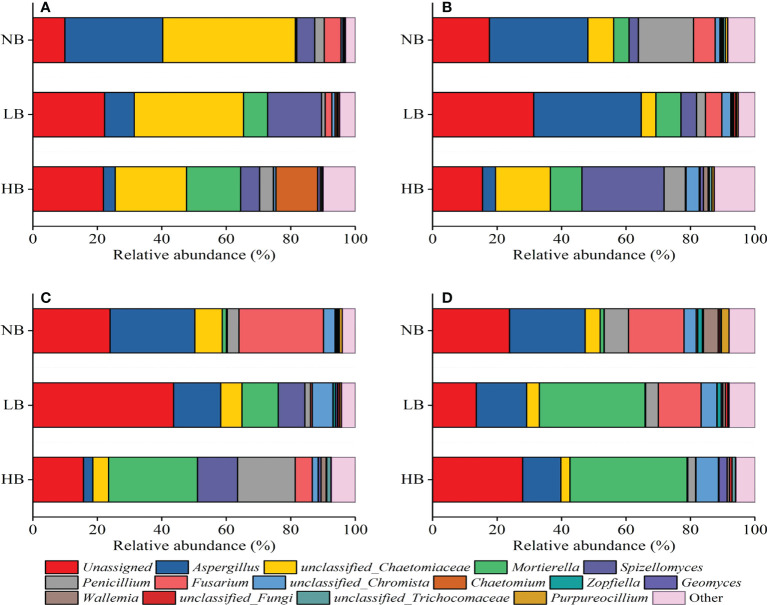
Effects of biochar on the relative abundance of fungi at the genus level in different soil depths: **(A)** At 0–10 cm, **(B)** 10–20 cm, **(C)** 20–30 cm, and **(D)** 30–40 cm.

At the genus level, the relative abundances of *Unassigned*, *Aspergillus*, *unclassified_Chaetomiaceae*, *Mortierella*, *Spizellomyces*, *Penicillium*, *Fusarium*, and *unclassified_Chromista* were higher (**Figure 4**). These fungi belonged to the dominant genera with a combined total of 75.40%–96.17%. Compared with NB treatment, the relative abundance of *Unassigned* fungi increased by 8.88% and 1.45% in LH and HB treatments, *Mortierella* increased by 12.96% and 20.71%, *Spizellomyces* increased by 5.28% and 8.81%, and *unclassified_Chromista* increased by 1.44% and 1.04%. The relative abundance of *unclassified_Aspergillus* decreased by 9.45% and 22.11%, *Penicillium* decreased by 5.39% and 0.02%, and *Fusarium* decreased by 8.65% and 12.44%, respectively.

It was also found that the application of biochar had a largely different influence on the relative abundance of the fungi in different soil depths, —for example, the relative abundance of *Aspergillus* in NB treatment was 8.31 (0–10 cm), 7.75 (10–20 cm), 9.03 (20–30 cm), and 2.00 (30–40 cm) times higher than that of HB treatment. The relative abundance of *Fusarium* in NB treatment was 2.58 (0–10 cm), 1.33 (10–20 cm), 48.65 (20–30 cm), and 1.30 (30–40 cm) times higher than that of LB treatment, while it was 62.44 (0–10 cm), 27.21 (10–20 cm), 5.03 (20–30 cm), and 218.93 (30–40 cm) times higher than that of HB treatment, respectively. In addition, LB and HB treatments significantly increased the relative abundance of *Mortierella* in 0–10 cm, which was 25.18 and 38.19 times higher than that of NB treatment. Moreover, the relative abundance of *Mortierella* in 30–40 cm was 28.58 and 31.61 times higher in LB and HB treatments than in NB treatment. The results also showed that LB and HB treatments significantly increased the relative abundance of *Chaetomium* in 0–10 cm by 0.29% and 12.71% compared with NB treatment.

### 3.6 Redundancy analysis of the soil fungal community structure and soil environmental factors

**Figure 5:**
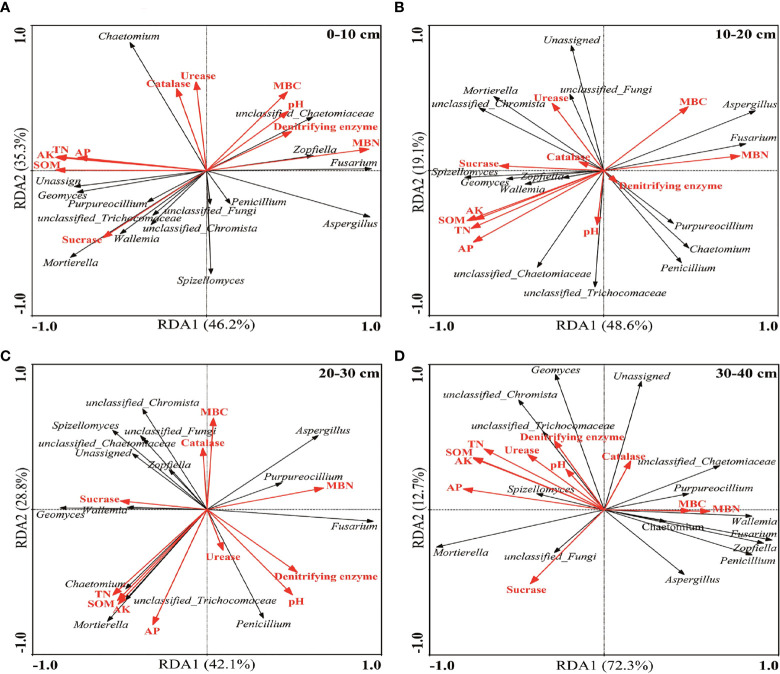
Redundancy analysis of fungi and chemical properties in different soil depths (genus): **(A)** At 0–10 cm, **(B)** 10–20 cm, **(C)** 20–30 cm, and **(D)** 30–40 cm.

The 15 dominant fungi in each soil depth were selected for RDA with the soil environmental factors (**Figure 5**). The results showed that MBN, TN, and AK were the main environmental factors affecting the structure of fungal communities in 0–10 cm, with a cumulative explanation rate of 81.5%. AK, AP, and TN were the main environmental factors affecting the structure of fungal communities in 10–20 cm, with a cumulative explanation rate of 67.7%. AK, TN, and SOM were the main environmental factors affecting the structure of fungal communities in 20–30 cm, with a cumulative explanation rate of 70.9%. AP, AK, and SOM were the main environmental factors affecting the structure of fungal communities in 30–40 cm, with a cumulative explanation rate of 85.0%.

## 4 Discussion

### 4.1 Effects of biochar on soil nutrient content and availability

Biochar is a carbon-rich material with stable properties, which is obtained by the pyrolysis of biomass under high-temperature and low-oxygen conditions (Mehdizadeh et al., 2021). The surface of biochar is rich in pores and has large charge density and specific surface area (Randolph et al., 2017). The special physicochemical properties of biochar make it an excellent soil amendment and offers great advantages in improving soil ecology and soil fertility (Zhu et al., 2017; Li et al., 2020). It was found that the application of biochar not only effectively increased the soil nutrient content but also improved soil pH and prevented soil acidification (Dai et al., 2017; Shi et al., 2019). Chagas et al. (2020) indicated that the physical structure of the soil was effectively improved, which helped to increase soil porosity and reduce soil capacitance after years of continuous application of biochar. In the present study, LB and HB treatments increased SOM, TN, AP, and AK at different soil depths, and HB treatment showed a higher improvement than LB treatment. There may be two main reasons for the increase in soil organic carbon after biochar application. On the one hand, the carbon in biochar exists mainly in an inert aromatic ring structure and has a high carbon content, so biochar application to the soil can directly increase the content of soil organic carbon (Han et al., 2021). On the other hand, biochar has strong adsorption properties, which can adsorb small organic molecules in the soil and promote the formation of soil organic matter from these molecules (Grunwald et al., 2017). Additionally, the porous structure of biochar provided attachment sites for the growth and reproduction of soil microorganisms, which indicated that biochar provides a good habitat for soil microorganisms (Raza et al., 2020). Lin et al. (2022) found that the increase in soil microorganisms accelerated the decomposition and release of organic nutrients from the soil, thus increasing the organic carbon content. The results of this study showed that SOM was hardly affected by soil depth after biochar application and was only slightly reduced (non-significant) in 20–40 cm. Our findings were also supported by Oladele et al. (2019), who observed that most of the SOM content remained in the topsoil (0–20 cm). Dong et al. (2016) also confirmed that biochar can improve the stability of SOM and help to enhance the carbon sequestration potential of the soil (Dong et al., 2016).

Nguyen et al. (2017) found that biochar had high C/N and bioactive substances, so when biochar was applied to the soil, the effectiveness of N would be reduced due to the effect of mineral N fixation by microorganisms. Differently, the results of the present study showed that the application of biochar (LB and HB treatments) significantly increased the TN content, and HB treatment showed a stronger improvement in the TN content. These results may be due to the fact that the biochar has little available nitrogen, which increases the nitrogen content in soil (Zhang et al., 2021). The application of biochar can also reduce N leaching losses and improve N fixation capacity (Lee et al., 2022). The experimental results also showed that the TN content of HB treatment in 10–40 cm decreased significantly with increasing soil depth, which indicated that the biochar could fix nitrogen in the topsoil (0–10 cm) and reduce the leaching of N from the lower soil layer. This may be related to the structural characteristics and high adsorption capacity of the biochar surface (Borchard et al., 2019). Oladele et al. (2019) confirmed that the increase of soil TN content was achieved by reducing N leaching and increasing N by microbial population. Biochar can significantly increase AP content, which may be due to the effectiveness and adsorptibility by influencing the anionic or microbial activity (Ukwattage and Lakmalie, 2022). Meanwhile, biochar can also effectively promote the dissolution, mineralization, and fixation of phosphorus by soil microorganisms, which, in turn, increase the AP content (Wu et al., 2021; Li and Li, 2022). Interestingly, AP did not migrate with increasing soil depth in this study, which implied that biochar enhanced the retention capacity of AP in the soil (Lehmann et al., 2011). Major et al. (2010) found that biochar was rich in soluble potassium, and the application of biochar could rapidly improve the AK content in the soil. In addition, Tian et al. (2021) and Xia et al. (2022) also confirmed that biochar could improve the soil microhabitat environment and accelerate the growth of phosphorus- and potassium-solubilizing bacteria, which eventually converted phosphorus and potassium from ineffective forms to effective forms. Notably, the experimental results also showed that biochar application significantly reduced the MBC content and showed a decreasing trend with increasing soil depth, which may be related to the higher rate of microbial N fixation than total N mineralization (Mcclellan et al., 2010; Dempster et al., 2011).

### 4.2 Effects of biochar on the diversity of soil fungal community structure

Soil microorganisms drive the material cycle in the soil, and the composition and structure of soil microbial communities have a strong influence on soil material cycling due to differences in their physiological characteristics (Ali et al., 2022b). Conversely, the physical structure and nutrient content of the soil also affect the community structure and composition of soil microorganisms (Simarani et al., 2018). As important members of soil microorganisms, fungi can promote the energy flow and material cycling in soil ecosystems and play an important role in soil–plant ecosystems (Joergensen and Wichern, 2008). Biochar can change the soil microbial community structure, but the change is influenced by the amount and timing of biochar application (Zhu et al., 2017; Yuan et al., 2019). PCA showed that the soil fungal community structure changed significantly after biochar application. Compared with NB treatment, the points showed obvious separation, especially in HB treatment. The results indicated that the more biochar was applied, the more significant changes in fungal community structure were observed, which was consistent with existing studies (Tarin et al., 2021). In addition, biochar can also affect the soil fungal community structure directly or indirectly. On the one hand, most fungi play the role of decomposer and have a higher utilization ability for carbon compared with bacteria (Wang et al., 2015). Meng et al. (2019) found that the addition of biochar improved the soil porosity and nutrient mineralization rate, which favorably promoted the increase in soil fungal abundance. On the other hand, biochar can indirectly promote the growth and reproduction of fungi by altering soil environmental factors such as SOM, TN, and AP (Liu et al., 2015; Zhu et al., 2017).

Fungi have a strong ability to decompose substances such as sugars and cellulose, which will increase the accumulation of soil organic matter (Jaiswal et al., 2017; Ali et al., 2022a). The application of biochar can change the microecological environment of the plant rhizosphere, improve the diversity of a fungal community, and enhance the disease resistance of crops (Kolton et al., 2016). In the present study, biochar application significantly increased the relative abundance of *Mortierella* (0–40 cm) and *Chaetomium* (0–10 cm). As a beneficial microorganism, *Mortierella* can strengthen the resistance of plants to pathogens and promote the growth of crops (Van Wees et al., 2008; Zheng et al., 2016). Franke-Whittle et al. (2015) found that *Chaetomium* can produce cellulase, xylanase, and laccase and also decompose cellulose and lignin. Previous studies have found that fungi were the most important pathogens in soils, and about 70% of plant diseases were caused by fungi,—for example, *Fusarium* can infect crop roots and destroy the vascular tissues of the root system, which, in turn, leads to serious soil-borne diseases (Jaiswal et al., 2017; Zhu et al., 2021). Yao et al. (2017) showed that applying 50–200 t·hm^-2^ of biochar significantly reduced the relative abundance of *Fusarium*. In the present study, the relative abundance of *Fusarium* was reduced by 8.65 and 12.44% in LB and HB treatments, which may be related to the allelopathy of phenolic acid in biochar (Wu et al., 2020; Bonanomi et al., 2021). Biochar application can affect not only the diversity of soil fungal communities but can also regulate the structure and composition of fungal communities, which could facilitate the evolution of fungi towards beneficial microorganisms (Bai et al., 2019). However, this effect may be closely related to the biochar type, application rate, and soil type (Khodadad et al., 2011). Therefore, the role and mechanisms of biochar in suppressing plant diseases still need to be studied in the future.

### 4.3 Effects of soil micro-ecological environment on the fungal community structure

Soil environmental factors are closely related to soil microorganisms, so analyzing the relationship between external environmental factors and fungal community structure will be beneficial to identify the key factors that affect the changes in a fungal community (Garcia-Pichel et al., 2013; Li et al., 2015; Kan et al., 2016). Zheng et al. (2016) found that biochar application changed the physical and chemical environmental conditions of the soil and affected the microbial habitat, which, in turn, changed the distribution of microorganisms and induced changes in the microbial community. In the present study, the results of RDA showed that the main environmental factors affecting the structure of fungal communities differed in different soil depths. In general, fungi prefer to grow in acidic environments. However, the application of biochar could improve soil pH, which may become the main factor affecting the soil microbial community structure (Nielsen et al., 2014; Chen et al., 2015). Conversely, Dai et al. (2016) found that the effect of soil nutrients on fungal communities was much greater than pH. Interestingly, AK was the main environmental factor affecting fungal community structure in all soil depths, which indicated that AK may play an important role in the regulation of soil fungal community structure by biochar in karst areas. The same viewpoint was confirmed in the studies of Dai et al. (2016) and Yao et al. (2017). Additionally, the influence of external environmental factors on the fungal community structure also differs with the biochar type, application time, and soil type (Muhammad et al., 2014; Li et al., 2019b). Therefore, the long-term effects of biochar on soil fungal community diversity should be strengthened in future studies, which can provide more references for the sustainable development of agriculture in karst areas.

## 5 Conclusion

Biochar application not only changed the nutrient content of yellow soil in different depths but also affected the diversity and structural composition of the fungal community. On the whole, the biochar dosage of 4.0% was the most effective in improving soil nutrients and regulating the structure of fungal communities. Meanwhile, biochar was conducive to improving the availability of nutrients in topsoil (0–20 cm), especially TN, AK, and MBN. However, biochar had more advantages in ameliorating microorganisms in deep soil (20–40 cm). To summarize, as a soil amendment, distiller’s grain biochar can be used to improve the soil fertility and microecological environment in karst areas.

## Data availability statement

The original contributions presented in the study are publicly available. These data can be found here: NCBI, PRJNA882202.

## Author contributions

MZ and JG designed the study and wrote the manuscript. MZ, YL, and QW performed the experiments. MZ, LL, and XG interpreted the results of the experiments and edited and revised the manuscript. JG approved the final version of the manuscript. All authors contributed to the article and approved the submitted version.

## Funding

This work was funded by the National Natural Science Foundation of China (no. 31860594 and no. 32060302), the Subsidy Project from NSFC of Guizhou Academy of Agricultural Sciences [(2021)32], and the Science and Technology Planning Project of Guizhou Province [(2020)1Y087].

## Acknowledgments

We would like to thank KetengEdit (www.ketengedit.com) for its linguistic assistance during the preparation of this manuscript.

## Conflict of interest

The authors declare that the research was conducted in the absence of any commercial or financial relationships that could be construed as a potential conflict of interest.

## Publisher’s note

All claims expressed in this article are solely those of the authors and do not necessarily represent those of their affiliated organizations, or those of the publisher, the editors and the reviewers. Any product that may be evaluated in this article, or claim that may be made by its manufacturer, is not guaranteed or endorsed by the publisher.
